# Open AccessLack of association between coat color abnormalities in Bali cattle (*Bos javanicus*) and the coding regions of the *MC1R* and *KIT* genes

**DOI:** 10.14202/vetworld.2023.1312-1318

**Published:** 2023-06-13

**Authors:** Jakaria Jakaria, Kholijah Kholijah, Sri Darwati, Qonita Rahman, Winni Liani Daulay, Ikhsan Suhendro, I. Made Londra, Mokhamad Fakhrul Ulum, Ronny Rachman Noor

**Affiliations:** 1 Department of Animal Production and Technology, Faculty of Animal Science, IPB University (Bogor Agricultural University), Bogor 16680, Indonesia; 2 Agricultural Technology Study Center (BPTP), JL. By Pass Ngurah Rai, Pesanggaran, Denpasar Selatan 80222, Bali, Indonesia; 3 Division of Reproduction and Obstetrics, School of Veterinary Medicine and Biomedical Sciences, IPB University (Bogor Agricultural University), Bogor 16680, Indonesia

**Keywords:** Bali cattle, coat color, KIT gene, melanocortin 1 receptor gene, single-nucleotide polymorphism

## Abstract

**Background and Aim:**

Coat color variations in cattle are known to be influenced by the melanocortin 1 receptor (*MC1R*) and receptor tyrosine kinase (*KIT*) genes. The presence of coat color abnormalities, such as white spots and albinism, in Bali cattle was the focus of this study. This study aimed to identify single nucleotide polymorphisms (SNPs) in the coding region of *MC1R* and exons 2 and 3 of *KIT* associated with coat color abnormalities in Bali cattle.

**Materials and Methods:**

The study included the analysis of 48 Bali cattle, including 20 individuals with standard coat color, 15 with white spots, and 13 with albinism. Total DNA was extracted using a DNA Extraction Kit, and *MC1R* (coding region) and *KIT* (exons 2 and 3) gene amplifications were analyzed using forward and reverse primers with polymerase chain reaction product lengths of 1071, 234, and 448 bp, respectively. The determination of *MC1R* and *KIT* gene diversity was analyzed through direct sequencing. Melanocortin 1 receptor and *KIT* gene sequence data were analyzed using BioEdit and MEGA6 to identify SNPs associated with standard and abnormal coat color phenotypes (white-spotted and albino) in Bali cattle.

**Results:**

No SNPs associated with coat color abnormalities were found in the coding region of *MC1R* and exons 2 and 3 of *KIT* genes in Bali cattle. However, the intron two regions of *KIT* contained the SNP g.70208534A>G, which showed a high degree of diversity. The AA genotype frequency was highest in albino Bali cattle, whereas the G allele frequency was highest and the A allele frequency was lowest in white-spotted Bali cattle.

**Conclusion:**

The results indicated that standard, white-spotted, and albino coat colors in Bali cattle could not be distinguished by analyzing the *MC1R* and *KIT* genes.

## Introduction

Coat color is a distinguishing feature of domestic cattle breeds and is controlled by genetic factors [1]. Pigmentation plays a crucial role in production and adaptability to environmental conditions, making coat color a critical factor in animal breeding [2]. The melanocyte-stimulating hormone (MSH) receptor is known to play a significant role in regulating coat color by facilitating the production of black (eumelanin) and red (phaeomelanin) pigments in melanocytes [3]. The MSH receptor genes carry three alleles, E^D^, E^+^, and e, with the E^D^ allele responsible for black coat color, whereas a frameshift mutation resulting in a prematurely terminated receptor is responsible for producing red coat color in homozygous *e/e* animals [2, 3].

Various genes, including *ASIP* (agouti), *TYR* (albinism), *TYRP1* (brown), *KIT* (dominant white), *KITLG* (roan), *PMEL* (dilution), melanocortin 1 receptor (*MC1R*) (extension), and *MITF* (white-spotted), have been identified as important pigmentation genes underlying coat color variation [2, 4–7]. The *MC1R* and *KIT* (KIT proto-oncogene, receptor tyrosine kinase) genes, which encode receptor tyrosine kinases [8], have been widely studied in cattle breeds, such as Japanese Brown [9], Italian beef and dairy [5], Sahiwal and Karan [2], and Zhoushan [10] cattle. The red and black coat color phenotypes of Angus cattle can now be easily determined [11]. In Bali cattle (*Bos javanicus*), the standard color is black for males and brown for females, with white stockings on their front and back legs and white markings on their hip [12]. However, inbreeding pressure in Bali cattle has led to the occurrence of coat color abnormalities, such as white-spotted and albino coats, which do not comply with the color standards of the breed. The incidence of white-spotted cases in Bali cattle has been reported to be 1.69% in Kupang, Province of Nusa Tenggara Timur, and 2.53% in the Bali Breeding Center, Province of Bali, whereas albino cases have been reported to be as high as 7.63% in Kupang [12, 13]. Albino Bali cattle, known as “Sapi Putih Taro,” are primarily found in the Taro District of Gianyar Regency, Bali Province, Indonesia [14]. The *MC1R* gene [15] and cytochrome oxidase subunit I gene within mitochondria [14] have been used to identify abnormalities in coat color, including white-spotting and albinism, in Bali cattle. Genetic markers based on the *MC1R* and *KIT* genes are crucial for identifying coat color in Bali cattle, as abnormal coat coloration results in noncompliance with the Indonesian National Standard [16].

This study aimed to identify single-nucleotide polymorphisms (SNPs) in the coding region of *MC1R* and exons 2 and 3 of *KIT* associated with coat color abnormalities (white-spotting and albinism) in Bali cattle using direct sequencing.

## Materials and Methods

### Ethical approval

This study was approved by the Animal Ethics Committee at Udayana University in Denpasar, Indonesia (Code ID: B/184/un14.2.9/pt.01.04/2021).

### Study period and location

This study was conducted from April 2022 to October 2022 at the Department of Animal Production and Technology, Faculty of Animal Science, IPB University, Bogor, Indonesia.

### Sample collection and DNA extraction

Blood samples (6 mL each) were collected from 48 Bali cattle (both male and female), including 13 albino cattle, 15 white-spotted cattle, and 20 cattle with the standard coat color. The Bali cattle with standard and white-spotted coat color were located at the Bali Cattle Breeding Center in Jembrana, Province of Bali, and the Breeding Center Unit in Serading, West Nusa Tenggara, Indonesia. Albino cattle were sampled at Taro Village, Regency of Gianyar, Province of Bali. Blood samples were collected through the jugular vein using a venoject containing ethylenediaminetetraacetic acid following the standard procedure. DNA extraction was performed using the Geneaid™ protocol (Geneaid Biotech Ltd., New Taipei city, Taiwan).

### Amplification and sequencing

The primers were designed to amplify exon 1, which was the target for the *MC1R* gene, and exons 2 and 3, which were the targets for the *KIT* gene (Table-1) [5, 13]. Polymerase chain reaction (PCR) amplification was conducted using GoTaq® Green Master Mix (PROMEGA Corporation, USA) with a total volume of 25 μL containing 25 pm/mL forward and reverse primer, 10−100 ng/μL DNA template, and nuclease-free water. The PCR reaction was performed on an Applied Biosystems GeneAmp PCR System 9700 (Thermo Fisher Scientific Inc. USA) with initial denaturation at 95°C for 5 min (1 cycle), denaturation at 95°C for 10 s (35 cycles), annealing for each target fragment (exon 1 for *MC1R*; exons 2 and 3 for *KIT*), and elongation at 72°C for 5 min (1 cycle). Polymerase chain reaction products from each fragment were visualized using electrophoresis in 1.5% agarose gel, stained with Floro Safe DNA (Axil Scientific Pte Ltd., Singapore), and documented using an ultraviolet transilluminator (AlphaImaginer, Alpha Inotech, Santa Clara, USA). The PCR products of *MC1R* and *KIT* were sent to 1^st^ BASE, Selangor, Malaysia, for sequencing.

**Table-1:** Primer sequence for *MC1R* gene and *KIT* genes.

Gene name	Position	Sequence (Forward and Reverse) (5’→3’)	Annealing (°C)	Size (bp)
*MC1R* *	Exon 1	TGA GAG CAA GCA CCC CTT C	59	1071
		TCA GGG ATG GTC TAG CCG A		
*KIT* **	Exon 2	TGT CGA GTA CAC AGA AGA TGG AA	59	234
		AAG TCC ACT TGA CAA ATC CTG GAC C		
	Exon 3	CTG CAG TGG AAG CAT TTG AC	62	448
		ACA CCC AGC AGA AAG CAA A		

* Matsumoto *et al*. [9];

** Fontanesi *et al*. [5], *MC1R*=Melanocortin 1 receptor

### Statistical analysis

The target sequences of *MC1R* and *KIT* were analyzed using FinchTV (https://www.softpedia.com/get/Science-CAD/FinchTV.shtml) and BioEdit 7.2 (https://bioedit.software.informer.com/) [17]. Single-nucleotide polymorphisms showing mutations at the sequence fragments of genes were identified through sequence alignment using Clustal W in MEGA version 10 (https://www.megasoftware.net/) [18]. The determination of genotype frequency, allele frequency, observed heterozygosity, expected heterozygosity, and Hardy–Weinberg equilibrium (HWE) was performed using POPGEN version 1.32 (https://sites.ualberta.ca/~fyeh/popgene_download.html) [19].

## Results and Discussion

**Figure-1:**
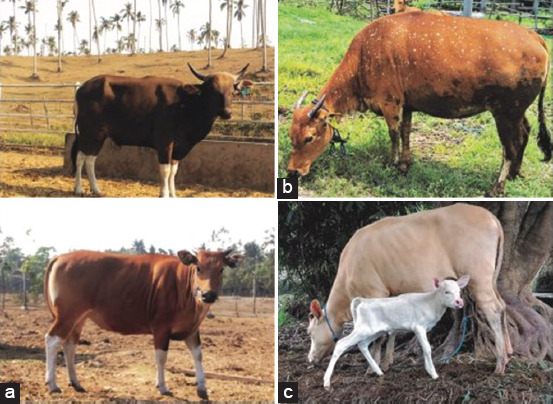
(a) Coat color variations in Bali cattle, including standard, (b) white-spotted, and (c) albino males and females.

**Figure-2:**
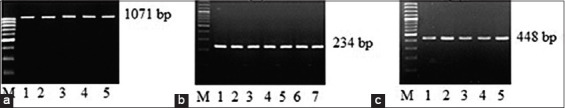
Electrophoresis results from the polymerase chain reaction (PCR) products of the melanocortin 1 receptor (*MC1R*) and *KIT* genes. (a) PCR products from *MC1R*, (b) *KIT* at exon 2, and (c) *KIT* at exon 3. M = 100 bp marker; lines 1–5/7 represent Bali cattle samples.

**Figure-3:**
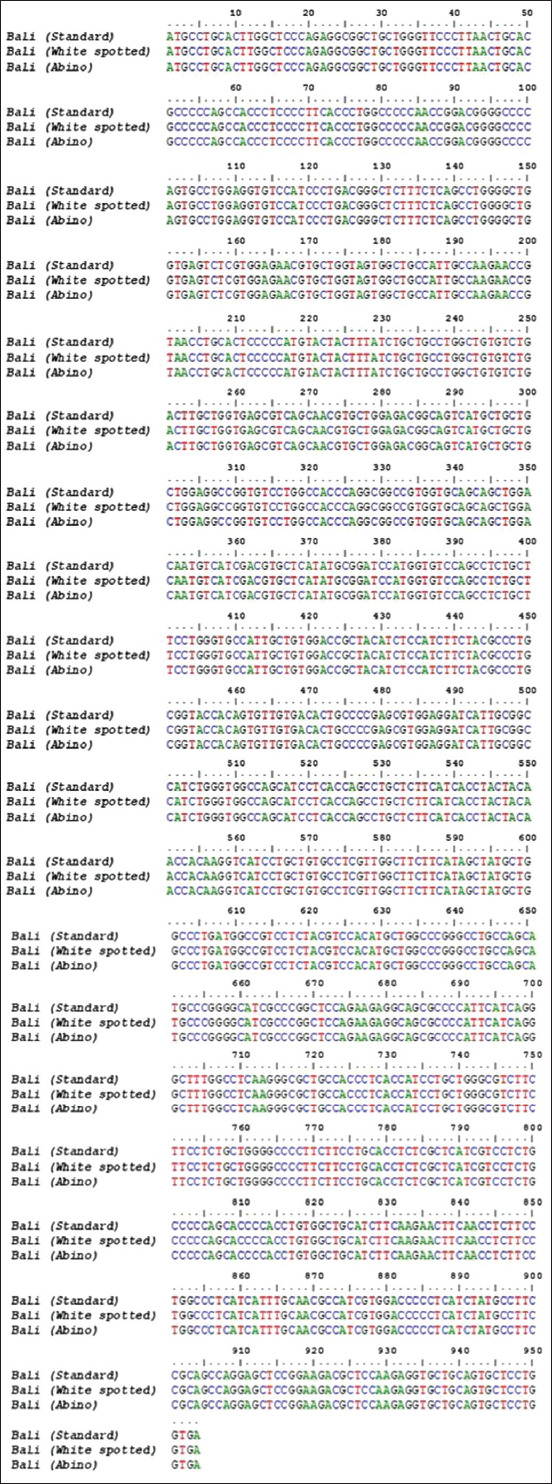
Nucleotide sequence alignment of the melanocortin 1 receptor gene coding region in Bali cattle samples, including standard, white-spotted, and albino cattle.

**Figure-4:**
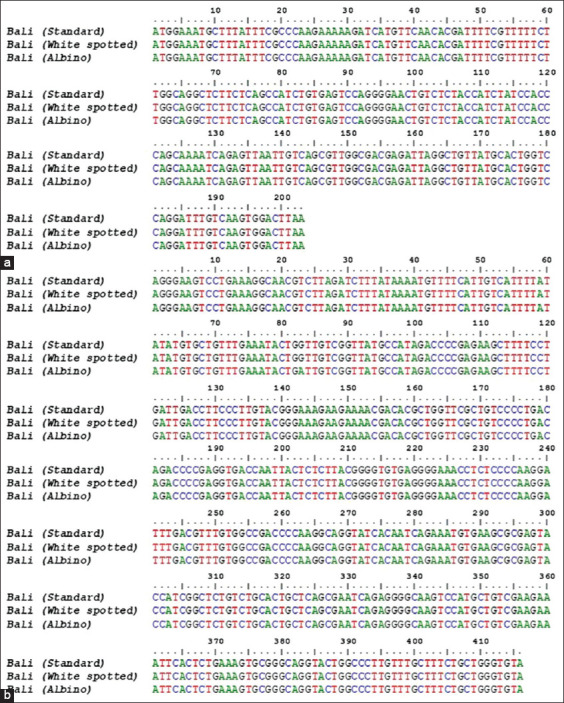
(a) Nucleotide sequences of exon 2 and (b) exon 3 of the *KIT* gene in Bali cattle with standard, white-spotted, and albino coat color.

**Figure-5:**
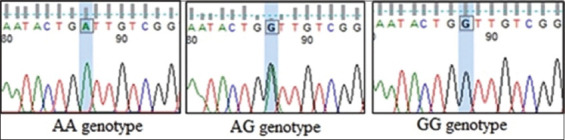
Determination of the genotypes of single nucleotide polymorphisms g.70208534A>G in the *KIT* gene exon 3 fragment.

The PCR products of the *MC1R* and *KIT* genes, corresponding to the target fragment (Figure-1) in male and female Bali cattle, were successfully amplified, including standard, white-spotted, and albino colors (Figure-2). No SNP or mutation was found in the coding region of *MC1R*, which was 954 bp in length from the initial codon (ATG) to the end codon (TGA), as shown in Figure-3. Exon 2 and 3 of the *KIT* gene fragment did not exhibit SNPs, but the SNP g.70208534A>G was found at the intron 2 position in the exon 3 fragment (Figure-4). This SNP showed three genotypes: GG, AG, and AA (Figure-5).

The g.70208534A>G SNP showed high diversity in all studied cattle (standard, white-spotted, and albino), but genotype AA was not detected in white-spotted Bali cattle (Table-2). Although standard Bali cattle did not show HWE status, white-spotted and albino Bali cattle did show HWE. However, the g.70208534A>G SNP could not distinguish coat color abnormalities, making it unsuitable as a molecular marker for coat color variation in Bali cattle.

**Table-2:** The frequency of genotype and allele in SNP g.70208534A >G in exon 3 of the *KIT* gene.

Phenotype	Genotype frequency			Allele frequency		Ho	He	χ^2^ test
	
	GG	GA	AA	G	A			
Standard (n = 20)	0.35	0.25	0.40	0.47	0.53	0.25	0.51	5.5
White spotted (n = 15)	0.47	0.53	0.00	0.73	0.27	0.53	0.40	1.7
Albino (n = 13)	0.12	0.25	0.63	0.46	0.54	0.58	0.52	0.2

SNP=Single nucleotide polymorphisms

**Figure-6:**
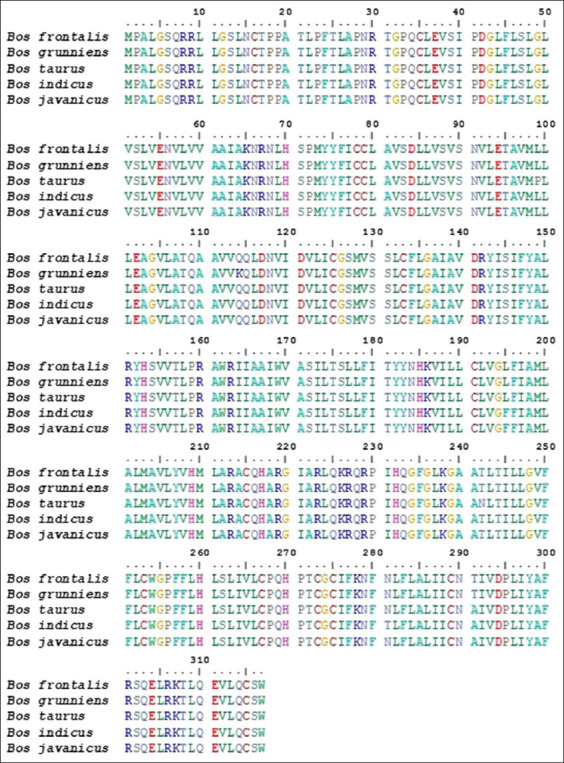
Amino acid sequence alignment of the melanocortin 1 receptor gene products of *Bos javanicus*, *Bos indicus*, *Bos taurus*, *Bos grunniens*, and *Bos frontalis*.

Bali cattle, native to Indonesia due to the domestication of *B. javanicus* [20], are known for their unique white coloration on their front and back legs and hips, with mature males being black and females brown [12]. We found that the *MC1R* and *KIT* genes in Bali cattle, including those with standard, white-spotted, and albino coat color, showed no difference in their nucleotide sequences. However, a novel SNP, g.70208534A>G, was detected in the intron 2 position of *KIT* at exon 3. Therefore, we further analyzed the profile of the estimated 317 amino acids in the product of the *MC1R* gene of Bali cattle (*B. javanicus*) (Figure-6) and compared the amino acid composition of this product with that of the *MC1R* gene products of *Bos indicus* (MG373575.1), *Bos taurus* (AF445641.1), *Bos grunniens* (FJ624478.1), and *Bos frontalis* (HM488960.1). We found discrepancies at positions L99P, Q114K, L195F, T242N, N281T, and T291A. Although there were differences in amino acid profile between *Bos* cattle groups, no specific amino acid composition was observed in Bali cattle (*B. javanicus*).

The E^D^ allele in the *MC1R* gene of *B. taurus* was previously found to have a single nucleotide substitution (SNP g.296.T>C) leading to a leucine to proline amino acid substitution at position 99 (L99P) [3]. However, this alteration was not observed in *B. javanicus*, *B. indicus*, *B. grunniens*, and *B. frontalis* (Figure-6). Other SNPs in *MC1R* have also been associated with coat color variations in other cattle breeds. For example, Japanese cattle have been found to possess the SNPs c.310G>- and c.871G>A, which produce brown coat color [9]. In contrast, Zhoushan cattle have the SNPs c.583T>C and p.F195L, as well as the non-synonymous SNP c.663, in *MC1R*, with the c.583T>C SNP leading to phenylalanine to leucine substitution at position 195 (F195L) [10]. Similarly, both Bali cattle (*B. javanicus*) and Sahiwal cattle (*B. indicus*) were found to have the nucleotide thymine (T) at a certain position, which codes for phenylalanine (F), as shown in Figure-6.

Single-nucleotide polymorphism g.72779776C>T and SNP g.72783182A>G found in exons 2 and 3 of *KIT*, respectively, have been associated with white-spotted coat color in Italian beef and dairy cattle [5], but they were not detected in Bali cattle in the present study. A novel SNP, g.70208534A>G, was found in the exon 3 fragment, specifically at intron 2 of the *KIT* gene, which was determined from the Ensemble genome (ENSBTAG00000002699). Interestingly, this SNP has not been previously reported in Ensemble, making this study the first to report its presence in *B. javanicus*. However, this new SNP was not able to differentiate between the coat color variations (standard, white-spotted, and albino) in Bali cattle. A HWE test revealed that SNP g.70208534A>G in the standard Bali cattle population was not at equilibrium, whereas the remaining showed an equilibrium state, which may be due to various factors, such as selection, mutation, genetic drift, and randomized mating in a large population [21].

## Conclusion

The findings of this study indicate that the coding region of the *MC1R* gene and exons 2 and 3 of the *KIT* gene are monomorphic in Bali cattle. Although a new SNP, g.70208534A>G, was identified in intron 2 of *KIT*, it could not distinguish between standard, white-spotted, and albino coat colors in Bali cattle. Thus, additional investigations using other gene fragments and a larger sample size are necessary to identify genetic markers that can accurately determine coat color in Bali cattle.

## Authors’ Contributions

JJ and RRN: Planned the research. JJ, IML, IS, and MFU: Collected the cattle blood samples. WLD, KK, and QR: Performed DNA isolation, PCR, and sequencing preparation. JJ, RRN, and SD: Conducted sequence analysis. JJ: Drafted the manuscript. All authors have read, reviewed, and approved the final manuscript.
